# Supplementary material for: A high-resolution mRNA expression time course of embryonic development in zebrafish
Source: eLife. 2017 Nov 16;6:e30860. doi: 10.7554/eLife.30860 (PMC5690287; doi:10.7554/eLife.30860)
Supplement: Supplementary file 6. [file elife-30860-supp6.zip › biolayout-clusters-files/Cluster088-genes.html]

Cluster088


# Cluster088: Genes

| Ensembl ID | Gene Name | Chr | Start | End | Biotype |
| --- | --- | --- | --- | --- | --- |
| ENSDARG00000098215 | BICD2 (1 of many) | 8 | 52965539 | 53002401 | protein\_coding |
| ENSDARG00000034093 | dclk2a | 1 | 36476298 | 36587895 | protein\_coding |
| ENSDARG00000026513 | hoxb6b | 12 | 27035779 | 27037408 | protein\_coding |
| ENSDARG00000040777 | inhbb | 9 | 37157499 | 37165084 | protein\_coding |
| ENSDARG00000101831 | irx1a | 16 | 737662 | 741827 | protein\_coding |
| ENSDARG00000074070 | irx5b | 25 | 35602496 | 35607765 | protein\_coding |
| ENSDARG00000068365 | lmx1bb | 8 | 33251003 | 33372681 | protein\_coding |
| ENSDARG00000074030 | myt1a | 23 | 30839955 | 30861401 | protein\_coding |
| ENSDARG00000053298 | nkx2.2a | 17 | 42775852 | 42783560 | protein\_coding |
| ENSDARG00000017673 | nova2 | 15 | 28753016 | 28833948 | protein\_coding |
| ENSDARG00000040946 | olig2 | 9 | 32940682 | 32942789 | protein\_coding |
| ENSDARG00000010144 | plppr3a | 2 | 26523462 | 26553791 | protein\_coding |
| ENSDARG00000070220 | pou3f2a | 4 | 5733163 | 5735887 | protein\_coding |
| ENSDARG00000001889 | tuba1a | 23 | 35609927 | 35612595 | protein\_coding |
